# Transforming the treatment of Alpha-Thalassemia: a single-center retrospective study on hematopoietic stem cell transplantation in transfusion-dependent pediatric patients

**DOI:** 10.1007/s00277-026-06855-y

**Published:** 2026-03-04

**Authors:** Lihua Huang, Zhenbin Wei, Gaohui Yang, Lianjin Liu, Zhongming Zhang, Hongwen Xiao, Zeyan Shi, Yibin Yao, Meiqing Wu, Lingyuan Pan, Wenqiang Xie, Zhaoping Gan, Beicai Liu, Zhongqing Li, Rui Huang, Xuemei Zhou, Yinghua Chen, Yanye Liu, Juan Bin, Zhaofang Pan, Huicheng Huang, Yongrong Lai, Rongrong Liu, Lingling Shi

**Affiliations:** 1 Department of Hematology, The First Affiliated Hospital of Guangxi Medical University, Nanning, Guangxi China; 2 NHC Key Laboratory of Thalassemia Medicine, Nanning, Guangxi China; 3 Guangxi Key laboratory of Thalassemia Research, Nanning, Guangxi China; 4 Guangxi Medical University, No.22, Shuangyong Road, Nanning, China

**Keywords:** Alpha-Thalassemia, Hematopoietic stem cell transplantation, Graft-versus-host disease, transfusion-dependent

## Abstract

Hematopoietic stem cell transplantation (HSCT) is the only definitive cure for transfusion-dependent α-thalassemia, though comprehensive studies on its effectiveness are limited. In this retrospective study, we analyzed the clinical characteristics of 21 pediatric patients with transfusion-dependent α-thalassemia who underwent HSCT, all of whom received a standardized conditioning regimen consisting of busulfan, cyclophosphamide, fludarabine, and anti-thymocyte globulin. After a median follow-up of 25 months (range: 7–92 months), the two-year overall survival (OS) and event-free survival (EFS) rates were both 90.2% (95% CI: 66.2–97.4%), and the two-year graft-versus-host disease-free, relapse-free survival (GRFS) rate was 82.3% (95% CI: 52.6–94.3%). The transplant-related mortality rate at two years was 5.0% (95% CI: 0.7–30.0%), with no cases of graft failure observed. Among the 19 surviving patients, hemoglobin levels significantly increased compared to pre-transplant levels (*p* < 0.05), and all became transfusion-independent. Hematopoietic stem cell transplantation is a curative treatment for α-thalassemia. For patients with transfusion-dependent α-thalassemia, HSCT should be performed as early as possible at an experienced transplant center when a suitable donor is available.

## Introduction

 Alpha-thalassemia affects approximately 5% of the global population, with a notably higher prevalence of 15% in Southeast Asia due to genetic predispositions [1, 2]. Recent global migration patterns have contributed to the spread of α-thalassemia to regions such as Northern Europe and North America [3]. Annually, around 56,000 infants worldwide are diagnosed with major forms of thalassemia, including over 13,500 cases of α-thalassemia, of which 5,500 result in neonatal fatalities due to severe disease manifestations [4]. Alpha-thalassemia is characterized by a deficiency in the synthesis of one to four α-globin genes. Mutations, either deletional or non-deletional, affecting three of these genes can result in hemoglobin H (HbH) disease. Due to the deficiency of α-chains, unstable tetramers of β-globin are formed. These tetramers are inefficient oxygen carriers, and precipitate causing hemolysis and ineffective hematopoiesis. Additionally, the instability of HbH disrupts the erythrocyte cell membrane, reducing red blood cell lifespan and resulting in various clinical manifestations, including moderate to severe hemolytic anemia, ineffective erythropoiesis, and splenomegaly [5, 6]. Patients with non-deletional forms of HbH disease, such as HbH Constant Spring, often experience more severe anemia and distinct clinical presentations compared to those with deletional forms [7]. These pathophysiological insights into α-thalassemia underscore the complexity of its clinical management and highlight the urgent need for further research into targeted therapies.

The management of transfusion-dependent α-thalassemia remains challenging due to a lack of comprehensive treatment reports. These challenges are exacerbated by the increasing severity of symptoms and the rising demand for transfusions as patients age [8], leading to chronic fatigue and significant impacts on both mental and physical health. Currently, HSCT offers a definitive cure for thalassemia major (TM), with event-free survival (EFS) rates for HLA-matched sibling donor transplants reaching up to 97% [9]. While HSCT has been extensively studied in patients with β-thalassemia major, there are limited studies focused on its application in patients with HbH disease who experience symptomatic anemia [10]. According to management recommendations from an international expert panel, young patients with thalassemia major who have an available HLA-identical sibling should be considered for HSCT as soon as possible [11]. In our study, we conducted a retrospective analysis of the clinical characteristics and outcomes of pediatric patients with transfusion-dependent α-thalassemia who underwent HSCT.

## Methods

### Patient characteristics

This was a single-center, retrospective study at the First Affiliated Hospital of Guangxi Medical University in China from March 2017 to May 2024 (Table 1). Inclusion criteria were: (1) a diagnosis of transfusion-dependent α-thalassemia, confirmed by hemoglobin electrophoresis, DNA analysis, and clinical blood transfusion dependence and (2) availability of matched sibling donor or alternative donor.

**Table 1:** Patient and transplant characteristics

Variables	Total *N* = 21
Genotype, *n* (%)	
--SEA/α^CS^α	13(61.9)
--SEA/αα^4.2^	1(4.8)
--SEA/-α^3.7^	1(4.8)
--/α^CS^α	4(19.0)
--/-α^3.7^	1(4.8)
αααα/αα	1(4.8)
Sex, *n* (%)	
Male	9(42.9)
Female	12(57.1)
Age, years, n (%)	
Median (range)	8(2–14)
Age < 7	6(28.6)
7 ≤ Age ≤ 15	15(71.4)
The age of first blood transfusion, month, n (%)	
Median (range)	8 (0–84)
0–24	17(81.0)
> 24	4(19.0)
Blood transfusion volume per session, U, n (%)	
1–2	15(71.4)
3–4	6(28.6)
Hepatomegaly, n (%)	9(42.9)
Splenomegaly, n (%)	19(90.5)
Regular iron chelation therapy	10(47.6)
Serum ferritin, ng/ml, n (%)	
≤1000	4(19.0)
1000–2500	13(61.9)
2500–5000† >5000	3(14.3) 1(4.8)
Cardiac T2*, ms, n (%)	
T2*>20^a^	14(66.7)
Hepatic LIC, mg/g dry weight, n (%)	
3< LIC ≤ 7^b^	4(19.0)
7< LIC ≤ 15^b^	2(9.5)
LIC ≥ 15^b^	6(28.6)
Donor type, n (%)	
Matched Sibling	9(42.9)
Alternative donor	12(57.1)
Graft type, n (%)	
BM^c^	3(14.3)
PBSCs^d^	5(23.8)
BM + PBSCs	10(47.6)
BM + CB^e^	3(14.3)

“Alternative donors” includes “Matched Unrelated Donors” and “Haploidentical Donors**”**

†The interval 2500–5000 includes both endpoints; all other intervals exclude the upper boundary.

^*a*^T2* spin-spin relaxation time; ^*b*^*LIC* liver iron content tested by magnetic resonance imaging; ^*c*^*BM* bone marrow; ^*d*^*PBSCs* peripheral blood stem cells; ^*e*^*CB* umbilical cord blood.

### Transplantation procedures

All patients received 20–30 mg/kg hydroxyurea orally once daily for 2–3 months before transplantation. The conditioning regimen for all patients included busulfan (Bu, 1 mg/kg i.v. 4x daily on days − 9 to -6), cyclophosphamide (Cy, 50 mg/kg i.v. once daily, days − 5 to -2), fludarabine (Flu, 50 mg/m² i.v. once daily from days − 12 to -10), and anti-thymocyte globulin (ATG, Thymoglobulin™, 2.5 mg/kg i.v. once daily, days − 4 to -1). Patients with matched sibling donors and haploidentical related donors received peripheral blood stem cells and bone marrow (PBSCs & BM), PBSCs, BM, or BM and cord blood (CB) grafts. Patients with matched unrelated donors received PBSCs grafts. All patients were given ganciclovir to prevent cytomegalovirus (CMV) infection. For GvHD prophylaxis, patients with matched sibling donors received cyclosporine (CsA), methotrexate (MTX), and mycophenolate mofetil (MMF). Patients with haploidentical related donors and matched unrelated donors received tacrolimus (Tac, 0.03 mg/kg/day), MTX (15 mg/m² on day + 1, and 10 mg/m² on days + 3, +6, and + 11), and MMF (250 mg/day for 90 days) [9]. For the treatment of acute GVHD (aGVHD), the first-line therapeutic drugs are glucocorticoids, with methylprednisolone being the most commonly used. The recommended starting dose is 1 mg·kg^− 1^·d^− 1^ or 2 mg·kg^− 1^·d^− 1^ (administered by intravenous injection in two divided doses). Meanwhile, adjust the trough concentration of CsA to 150–250 µg/L or tacrolimus to 5–15 µg/L, and evaluate the efficacy of glucocorticoids in a timely manner. If the hormones are ineffective or there is hormone dependence, on the basis of maintaining the effective concentration of calcineurin inhibitors (CNI), add second-line drugs such as anti-interleukin-2 receptor antibody (IL-2RA) monoclonal antibodies (basiliximab, ruxolitinib), and evaluate the efficacy in a timely manner [12]. 

### Definitions and endpoints

Neutrophil engraftment was defined as the first of 3 consecutive days with a neutrophil count ≥ 0.5 × 10^9^/L in peripheral blood without G-CSF (Granulocyte Colony Stimulating Factor). Platelet engraftment was defined as platelet count of ≥ 20 × 10^9^/L without transfusion support for 7 consecutive days. Primary graft failure was defined as a lack of evidence of engraftment or hematological recovery of donor cells within the first month after transplantation and transfusion dependence. In contrast, secondary graft rejection was defined as cytopenia after the initial engraftment with donor chimerism of less than 5% and a return to a thalassemic bone marrow or bone marrow hypoplasia requiring red cell transfusion. GVHD was classified by the Glucksberg and NIH classifications [13, 14]. The patients who had never encountered any sign of chronic GVHD (cGVHD) or had a history of completely resolved cGVHD were recorded as GVHD-free. Deaths without relapse of TDT was considered transplant related mortality (TRM). The primary end-points were overall survival (OS), thalassemia-free survival (TFS), and thalassemia-GVHD-free survival (TGFS). OS was defined as the time from the date of transplantation until death from any cause or the last follow-up. EFS was defined as time from transplantation to death, GF or recurrent TDT. TGFS was defined as being alive without thalassemia and GVHD.

## Statistical analyses

We summarized continuous variables using number of patients, and median (IQR). We summarized categorical variables using counts and percentages. In the primary analysis, we use two-sided exact 95% CIs using the Kaplan-Meier method. SPSS v27.0 and R v4.5.0 were used for the analyses.

## Results

The pre-transplant and transplant characteristics of the patients are summarized in Table 1.

Between January 1, 2017, and July 16, 2024, a total of 21 patients were included in this study. The median age at transplantation was 8 years (range: 2–14 years), and 12 (57.1%) patients were female. Among the cohort, 20 (95.2%) patients were diagnosed with HbH disease, with 17 (81.0%) carrying non-deletional α-thalassemia and 3 (14.3%) carrying deletional α-thalassemia, while one (4.8%) patient had gene quadruplications. The genotypic distribution of the cohort included the following: --SEA/α^CS^α (61.9%), --/α^CS^α (19.0%), --SEA/αα^4.2^ (4.8%), --SEA/-α^3.7^ (4.8%), --/-α^3.7^ (4.8%), and αααα/αα (4.8%). Notably, one patient with the genotype --SEA/α^CS^α had previously undergone hypospadias surgery, and the patient with αααα/αα also presented with β-thalassemia trait (17 M/N). The median age at first blood transfusion was 8 months (range: 0–84 months), with 11 (52.4%) of the patients receiving irregular transfusions. With age, transfusion frequency rose in all patients, reaching once monthly; prior annual rates of 3, 4, 5 and 6 transfusions were seen in 3, 4, 1 and 3 cases, respectively. Among the 28.6% who eventually required 3–4 units of blood transfusions per month, pre-transfusion hemoglobin levels between 6 and 9 g/dL. Extramedullary hematopoiesis was observed in 19 (90.5%) patients, with 19 (90.5%) presenting splenomegaly and 9 (42.9%) presenting hepatomegaly. None of the patients had undergone splenectomy. 10 (47.6%) patients received regular iron chelation therapy. Among 14 patients who underwent magnetic resonance imaging (MRI) assessments of the heart and liver, all had cardiac T2*>20 ms, while 6 (42.9%) patients had a liver iron concentration (LIC) ≥ 15 mg/g dry weight. According to previous studies, myocardial iron content cannot be predicted from serum ferritin or liver iron, because the heart takes up and releases iron more slowly than the liver, so cardiac iron accumulation lags behind [15, 16]. Patients received hematopoietic stem cell grafts from either matched sibling donors or alternative donors, based on donor availability. Matched sibling donors accounted for 9 (42.9%) cases while alternative donors included 12 (57.1%) cases. Among the alternative donors, 8 (38.1%) were matched unrelated donors (10/10 or 9/10 HLA-A, B, C, DR, and DQ loci) [8], and the remainder were haploidentical related donors.

The median follow-up duration after transplantation was 25 months (range: 7–92), with the last follow-up conducted on December 16, 2024. All patients successfully achieved primary engraftment (Table 2).

**Table 2:** Transplantation outcomes

Variables	Total *N* = 21
Overall survival, n	19
2-year OS^1^ (95% CI^2^)	90.2% (66.2–97.4)
Event-free survival, n	19
2-year EFS^3^ (95% CI)	90.2% (66.2–97.4)
Graft-versus-host disease-free, relapse-free survival, n	18
2-year GRFS^4^	82.3% (52.6–94.3)
Transplant related mortality, n	1
2-year TRM^5^ (95% CI)	5.0% (0.7–30.0)
Mix chimerism	1(4.8%)
GF^6^	0(0%)
Neutrophil graft, days, median (IQR^7^)	12(11–13)
Platelet graft, days, median (IQR)	14(12–16)
Infection	19(90.5%)
VOD/SOS^8^	0(0%)
HC^9^	7(33.3%)
PRES^10^	0(0%)
aGVHD^11^, n	6
Cumulative incidence (95% CI)	28.9% (14.1–54.3)
Grades 2–4 aGVHD^11^, n	2
Cumulative incidence (95% CI)	10.5% (2.7–35.9)
Grades 3–4 aGVHD^11^, n	2
Cumulative incidence (95% CI)	11.3% (2.9–38.6)
cGVHD^12^, n	4
Cumulative incidence (95% CI)	36.5% (14.5–72.3)

^*1*^ *OS* overall survival; ^*2*^ *CI* confidence interval; ^*3*^ *EFS* event-free survival; ^*4*^ *GRFS* graft-versus-host disease-free, relapse-free survival; ^*5*^ *TRM* transplant related mortality; ^*6*^ *GF* graft failure; ^*7*^ *IQR* Interquartile range; ^*8*^ *VOD* hepatic veno-occlusive disease/ sinusoidal obstruction syndrome; ^*9*^ *HC* hemorrhagic cystitis; ^*10*^ *PRES* posterior reversible encephalopathy syndrome; ^*11*^ *aGVHD* acute graft versus host disease at 100 days; ^*12*^ *cGVHD* chronic graft versus host disease at 2 years.

**Fig. 1:**
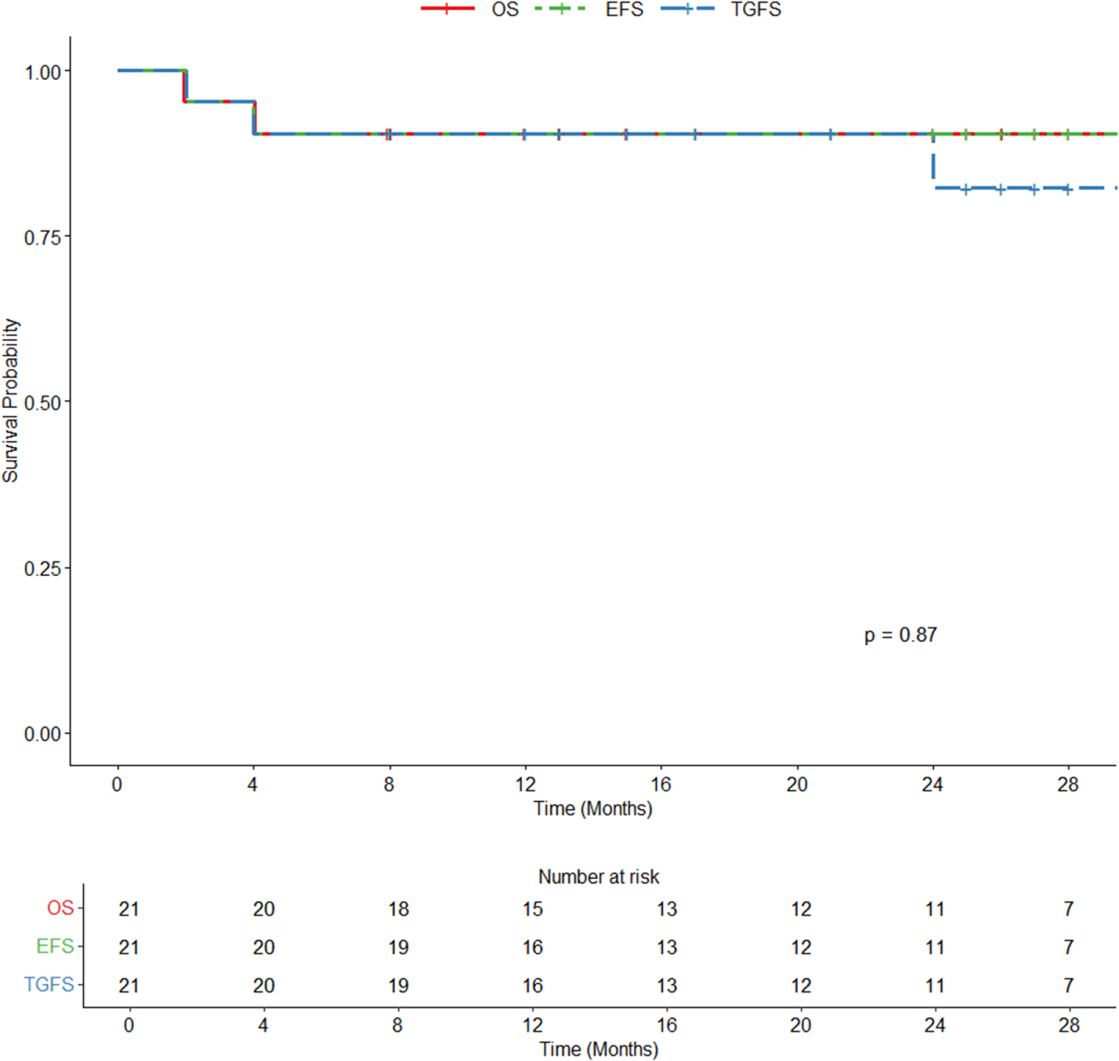
The 2-year probabilities of OS、EFS、TGFS for patients

The median time to neutrophil and platelet engraftment was 12 days (IQR: 11–13) and 14 days (IQR: 12–16). The incidence of aGVHD was 28.6%, with grades 2–4 aGVHD and grades 3–4 aGVHD observed in 9.5% of cases each. Two patients developed severe (grades 3–4) aGVHD: one experienced grade 4 gastrointestinal involvement and grade 3 skin rash, while the other had grade 4 gastrointestinal involvement. Unfortunately, two patients died during the follow-up period. One patient developed grades 3–4 aGVHD and ultimately succumbed to hypoxemia caused by severe pneumonia, while another patient died from an accidental fall 56 days post-transplant. The two-year transplant-related mortality (TRM) rate was 5.0%. cGVHD occurred in four patients. One patient developed mixed chimerism that persisted for 14 months. The lowest chimerism level was 56.43%, and the nadir hemoglobin was 6.2 g/dL; mean hemoglobin during this period was 10.3 g/dL. After re-infusion of donor stem cells, the chimerism ultimately rose to 99%. Infections were the most common post-transplant complications, affecting 19 (90.5%) of patients. Five patients developed pneumonia, one of whom died from severe pneumonia, while another patient was diagnosed with Klebsiella pneumoniae infection but recovered following treatment. Six patients received letermovir prophylaxis post-transplantation. Of the seven patients who experienced cytomegalovirus (CMV) reactivation, five had not received letermovir. All cases of CMV reactivation were successfully treated with ganciclovir, gamma globulin, or foscarnet sodium. Reactivation of Epstein-Barr virus (EBV) was observed in 3 (14.3%) of patients. Hemorrhagic cystitis (HC) occurred in 33.3% of cases. The two-year OS, EFS, and GRFS rates were 90.2%, 90.2%, and 82.3% (Fig. 1). Notably, all surviving patients became transfusion-independent by the end of follow-up.

## Discussion

HbH disease presents in two primary forms: deletional and non-deletional. The more common deletional form arises when two α-globin genes are deleted on one chromosome and a single α-globin gene is deleted on the other (--/-α). This form exhibits a range of clinical symptoms, which vary depending on the severity of the imbalance in globin chain synthesis. In contrast, the less common non-deletional form occurs when two α-globin genes are deleted on one chromosome and a pathogenic mutation affects an α-globin gene on the other (--/α^ND^α). This mutation frequently introduces a termination codon that extends the α-globin chain by 31 amino acids, producing an unstable hemoglobin variant known as Hb Constant Spring (Hb CS) [17]. In our study, one patient carried the genotype αααα/αα and co-inherited β-thalassemia (17 M/N). Such triplications or quadruplications of the α-globin gene, when combined with β-thalassemia variants, can lead to more severe clinical phenotypes [18]. A thorough understanding of each specific genotype and its associated clinical manifestations is essential for optimizing transplant outcomes and managing post-transplant care. It is particularly critical in predicting and mitigating potential complications, such as graft-versus-host disease or transplant rejection, to improve long-term prognosis.

Our study characterized the transfusion patterns and associated hematological parameters in patients with transfusion-dependent α-thalassemia. First, as detailed in Table 1, most patients began transfusions early in life, with 81% receiving their first transfusion before the age of two. The earliest transfusion was administered shortly after birth, while the latest occurred at the age of seven. Previous studies have shown that patients with non-deletional α-thalassemia required transfusions significantly earlier (mean age: 1.5 ± 2.1 years) compared to deletional α-thalassemia patients (mean age: 11 ± 5.5 years). Moreover, non-deletional patients had a lower mean hemoglobin level of 7.2 g/dL (range: 3.8–8.7 g/dL) in infancy compared to their deletional counterparts, who exhibited a higher mean hemoglobin level of 8.5 g/dL (range: 6.9–10.7 g/dL) [6, 19]. Second, prolonged transfusion therapy often resulted in significant iron overload. In our cohort, 85.7% of patients showed varying degrees of hepatic iron overload on MRI evaluation. Even in the absence of regular red blood cell transfusions, patients with HbH disease frequently experience iron overload due to increased intestinal iron absorption, which can cause severe organ damage over time [10]. Beyond its physiological impact, iron overload also imposes a substantial financial burden due to the high cost of iron-chelation therapies. Third, growth and development were notably impaired in many patients. When compared to standardized growth charts for children and adolescents aged 0 to 18 years in China [20], 85.7% of patients displayed growth and developmental delays, comprising 14.3% with deletional and 66.7% with non-deletional genotypes. These delays became increasingly evident during adolescence—a stage that demands higher hemoglobin levels to meet critical developmental needs. Even though 28.6% of patients eventually needed 3–4 units every month, their post-transfusion hemoglobin never rose high enough to meet the demands of growth, development or physical activity [21]. 

These findings underscore the importance of timely and optimized management of transfusion-dependent α-thalassemia, not only to mitigate the complications associated with transfusion and iron overload but also to address the long-term impact on growth and quality of life.

Historically, splenectomy has been a key intervention in managing HbH disease, often providing a short-term increase in hemoglobin levels of 10–20 g/L [22]. However, splenectomy carries significant risks, including severe complications such as infections, sepsis, thrombosis, and heart failure [23]. These risks are particularly pronounced in children under the age of five, for whom splenectomy is generally contraindicated due to the elevated likelihood of life-threatening complications [24]. In recent years, HSCT has emerged as a curative option for α-thalassemia, offering the potential for long-term remission or even a complete cure. Our study highlights the clinical benefits of HSCT in patients with transfusion-dependent α-thalassemia, demonstrating that this treatment can lead to significant improvement in clinical outcomes and transfusion independence. By analyzing the clinical characteristics of these patients, we have identified key patient profiles likely to derive the greatest benefit from HSCT. However, our study also faced certain limitations, including a relatively small sample size and OS and EFS rates that, while promising, did not exceed those observed in β-thalassemia patients treated at our center [9]. These limitations underscore the need for larger-scale studies to confirm and expand on our findings.

In conclusion, this study represents significant progress in understanding the role of HSCT in treating transfusion-dependent α-thalassemia. Most of the patients included in this research carried non-deletional or mixed-deletion genotypes and presented with severe anemia from an early age, transfusion dependence, growth delays, and extensive extramedullary hematopoiesis. HSCT provides an effective curative solution for such patients, particularly in cases where transfusion therapy alone fails to meet clinical needs. The limitations of this study include its single-center design, small number of cases, and the absence of statistical comparisons across α-thalassemia genotypes, all of which restrict the strength of the conclusions that can be drawn. Moving forward, it will be critical to expand research on the long-term efficacy and safety of HSCT in α-thalassemia, refine patient selection criteria, optimize transplantation protocols to improve outcomes, and minimize associated risks. These efforts will be essential for broadening access to this potentially life-saving treatment for α-thalassemia patients worldwide.

## Data Availability

The study protocol, statistical analysis, plan are available from the corresponding author on a reasonable request. Requests to access these datasets should be directed to rong2liu@hotmail.com.

## Data Availability

The study protocol, statistical analysis, plan are available from the corresponding author on a reasonable request. Requests to access these datasets should be directed to rong2liu@hotmail.com.
